# Demorest’s Monthly

**Published:** 1873-08

**Authors:** 


Demorest's Monthly Magazine for September is rich in literary novelties, and also gives a fine display of the new fall fashions. Demorest seems to outbid all his cotemporaries in the value of premiums to his subscribers; he announces an astounding offer for 1874, of the large and celebrated chromo, "The Old Oaken Bucket," after Jerome Thompson, and several over equally large and valuable chromos, "The Captive Child," "Home, Sweet Home," and "After the Storm," for the ensuing three years, worth fifteen dollars each. This is certainly unparalleled, and we wonder how it can be done. Send for circular. Address, W. Jennings Demorest, 838 Broadway, New York.

